# Association between vitamin D status and thyroid cancer: a meta-analysis

**DOI:** 10.3389/fnut.2024.1423305

**Published:** 2024-06-18

**Authors:** Yue Hu, Chongxiang Xue, Shumeng Ren, Lishuo Dong, Jiaqi Gao, Xiuyang Li

**Affiliations:** ^1^Institute of Metabolic Diseases, Guang'anmen Hospital, China Academy of Chinese Medical Sciences, Beijing, China; ^2^Qi-Huang Chinese Medicine, Beijing University of Chinese Medicine, Beijing, China; ^3^Graduate School, Beijing University of Chinese Medicine, Beijing, China; ^4^Department of Integrative Oncology, China-Japan Friendship Hospital, Beijing, China; ^5^Changchun University of Chinese Medicine, Changchun, Jilin, China; ^6^Guang'anmen Hospital, School of Clinical Medicine, Beijing University of Chinese Medicine, Beijing, China

**Keywords:** vitamin D, meta-analysis, thyroid cancer, vitamin D deficiency, 25 (OH)D, 1, 25 (OH)D

## Abstract

**Background:**

Cumulative evidence has suggested that vitamin D deficiency is related with an increased susceptibility to various types of cancers. However, the association between vitamin D and thyroid cancer (TC) has remained to be unknown. Thus, there has been an urgent need for a meta-analysis to summarize existing evidence on vitamin D levels and the risk of TC.

**Objective:**

This meta-analysis aimed to figure out the association between vitamin D level and the risk of TC.

**Methods:**

A systematic search was performed for eligible articles on the association between vitamin D and TC based on PubMed, Embase, Web of Science, Cochrane, and ClinicalTrials.gov. Outcomes were the vitamin D level of cases with TC and the incidence of vitamin D deficiency in cases with TC comparing with the controls. The effect measures included standardized mean difference (SMD), ratio of means (RoM), and odds ratio (OR). A dose-response meta-analysis was performed to assess the correlation between vitamin D level and the risk of TC. Subgroup analyses and meta-regressions were conducted to explore the source of heterogeneity. And publication bias was evaluated through Begg's and Egger's tests.

**Results:**

Results of the meta-analysis revealed lower levels of vitamin D in TC cases comparing with those in control [SMD = −0.25, 95% CI: (−0.38, −0.12); RoM = 0.87, 95% CI: (0.81, 0.94)] and the levels of 1,25 (OH)D in cases with TC were also lower than controls [SMD = −0.49, 95% CI: (−0.80, −0.19); RoM = 0.90, 95% CI: (0.85, 0.96)]. And vitamin D deficiency was associated with the increased risk of TC [OR = 1.49, 95% CI: (1.23, 1.80)]. Additionally, results from the dose-response meta-analysis showed that there is a 6% increase in the risk of TC for each 10 ng/ml decrease in 25 (OH)D levels [OR = 0.94; 95% CI: (0.89, 0.99)].

**Conclusions:**

Individuals with TC had lower levels of vitamin D compared to controls, and vitamin D deficiency was correlated with an increase risk of TC.

**Systematic review registration:**

https://www.crd.york.ac.uk/PROSPERO/display_record.php?RecordID=504417, identifier: CRD42024504417.

## 1 Introduction

Thyroid cancer (TC) is the most common malignancy of the endocrine system, encompassing three overarching histological classifications: differentiated, medullary and anaplastic thyroid cancer (1, 2). Extensive research indicates a consistent upward trend in the worldwide prevalence of TC over the past few decades, while its mortality rates have remained relatively stable (3–9). The global age-standardized incidence rates of TC in 2020 were found to reached 3.1 per 100,000 person-years in males and 10.1 per 100,000 person-years in females. However, the mortality rates have remained to be 0.5 per 100,000 person-years worldwide over the past four decades (10, 11). GLOBOCAN 2020 database, compiled by the World Health Organization, has speculated that TC would become the second most prevalent cancer in women and the ninth in men by 2030, severely aggravate the burden of economic and health for society. (3). Thus, the prevention, diagnosis and management of TC have been an emerging problem (10, 12–16).

Vitamin D is a versatile steroid pro-hormone that is predominantly synthesized in the skin through the sunlight exposure and partly obtained from dietary sources (17). Both cutaneous and dietary vitamin D will be metabolized into 25-hydroxyvitamin D [25 (OH)D] in the liver and further converted into 1, 25-hydroxyvitamin D [1,25 (OH)D], the bio-active form of vitamin D, in the kidneys (18). The classical roles of 1,25 (OH)D include mediating calcium absorption in the intestine, regulating bone metabolism and so on (18). Recently, accumulating evidence has highlighted the potential anticancer action of vitamin D through its ability to hinder the proliferation, invasiveness and metastatic potential of malignant cells and promoting cell differentiation. In light of those mechanisms above, it is widely believed that the deficiency of vitamin D may be closely associated with an increased susceptibility to various types of cancers.

Also, the relationship between TC and vitamin D levels has been a topic of debate (19). Numerous clinical and experimental studies have suggested that individuals with TC tend to exhibit lower levels of vitamin D compared to both healthy individuals and those with benign thyroid nodules, while findings in some other studies have presented contrasting results (20–22). As a result, the association between vitamin D deficiency and the risk of TC has also remained inconclusive so far (23).

Given those aforementioned circumstances, there is an urgent need for a meta-analysis to summarize existing evidence quantitatively on vitamin D levels and the risk of TC. Although a few related meta-analyses have discussed the elevated risks of TC in individuals with vitamin D deficiency compared to those with sufficient vitamin D levels, whether these risks differ by the severity of the deficiency and how different forms of vitamin D are associated with TC still remained unclear (24, 25). Consequently, we performed this meta-analysis to address these uncertainties in more comprehensive precise manners.

## 2 Research design and methods

### 2.1 Search strategy

A systematic search was conducted for relevant published articles from five databases (PubMed, Embase, Web of Science, Cochrane, and ClinicalTrials.gov) based on medical subjects heading terms, key words and word variants for “thyroid cancer,” “thyroid carcinoma,” “thyroid neoplasm,” “vitamin D,” “25-hydroxyvitamin D,” “25 (OH)D,” “25 OHD” and “cholecalciferol”. Additional published studies were retrieved by screening the references of the relevant original reports. A further search in preprint servers including Biorxiv and Medrxiv for preprint articles were conducted in order to identify eligible unpublished literature. All searches were performed on November 29th, 2023 and a more specific search strategy is shown in Table 1.

**Table 1:** Search strategy in PubMed.

**#**	**Term**
#1	(vitamin D[Title/Abstract]) OR (25-hydroxyvitamin D[Title/Abstract]) OR (25 (OH)D[Title/Abstract]) OR (25 OHD[Title/Abstract]) OR (cholecalciferol[Title/Abstract]) OR (“vitamin d”[MeSH Terms])
#2	(thyroid cancer[Title/Abstract]) OR (thyroid neoplasm[Title/Abstract]) OR (thyroid tumor[Title/Abstract]) OR (thyroid carcinoma[Title/Abstract]) OR (carcinoma of thyroid[Title/Abstract]) OR (thyroid neoplasm[MeSH Terms])
#3	#1 AND #2

### 2.2 Study selection

Inclusion criteria were as follows: (1) study type: prospective or retrospective observational designs including case-control studies, cohort studies and cross-sectional studies (2) population: patients diagnosed with thyroid cancer and health controls or cases with benign thyroid disease (3) study content: articles assessing the association between vitamin D and thyroid cancer which are published or unpublished (4) outcome: the levels of vitamin D in TC groups and control groups (healthy cases or those with benign thyroid diseases), the link between vitamin D deficiency and the risk of TC which was always expressed as odds ratios with 95% confidence intervals (CIs).

The exclusion criteria were defined as follows: (1) case reports, reviews, letters, conference records (2) studies conducted on animals (3) studies based on public databases or other non-original data.

Two investigators independently performed the three-step selection process consisting of title-screening, abstract-screening and full-text screening. Disagreement between investigators (Yue Hu and Shumeng Ren) were solved by consensus with a third person (Lishuo Dong).

### 2.3 Data extraction and quality assessment

The following characteristics were extracted from eligible studies: title, the name of the first author, year of publication, country where the study was conducted, study design, the type of thyroid cancer, the source of control, sample size of patients and controls, age, sex, body mass index, dietary habits, and tobacco use of participants, timing of vitamin D exposure measurement, methods of vitamin D measurement and the type of vitamin D measured. The following outcomes of studies were also extracted: levels of vitamin D in both TC cases and controls, adjusted odds ratios (OR) with 95% confidence intervals (CIs) for every category of vitamin D levels, the distribution of cases and controls in each category, variables adjusted for in the analysis, and the number of patients and controls with sufficient vitamin D levels, as well as the number of participants with vitamin D deficiency in each group. Data were extracted by two investigators independently using a standardized data extraction form, and any discrepancy was resolved by a cross-check.

The nine-star Newcastle-Ottawa quality assessment scales were used to assess the quality of case-control and cohort studies, while the scale of Agency for Healthcare Research and Quality (AHQR) were applied to cross-sectional studies. Two investigators (Yue Hu and Shumeng Ren) assessed the quality of studies independently, any discrepancy were resolved by consensus with a third person (Lishuo Dong).

### 2.4 Data synthesis and analysis

The primary outcome was the level of vitamin D in patients with thyroid cancer, whose effect size was reported in terms of standardized mean difference (SMD) with 95% CIs, calculated from the mean value and standard deviation (SD). Methods by Luo et al., and Wan et al. were used to optimally estimate the mean and SD from the sample size, median, mid-range and mid-quartile range when only five-number summary was provided by included articles (26, 27). And a subgroup analysis were conducted based on the methods of vitamin D measurement, the timing of measurement, the countries, the sample source of vitamin D and the source of controls. Meta-regressions were performed on the basis of the methods of vitamin D measurement, the timing of measurement, the countries, the sample source of vitamin D, the source of controls, the publication year, the sex ratio of participants and the age of participants. To provide clinicians with more intuitive clinical interpretation of the differences of vitamin D levels between case group and control group, the ratio of means (RoM) with 95% CIs, defined by the mean value in the case group divided by the mean value in the control group, was also introduced as another effect size. The second outcome was the incidence of vitamin D deficiency in patients with TC presented by odds ratio (OR) with 95% CIs. Random effects models were used to ensure the robustness of these results. A subgroup analysis and a meta-regression were conducted based on the timing of measurement and the source of the controls.

A dose-response meta-analysis for a deeper explanation of correlation between the vitamin D level and the risk of TC was also conducted. Firstly, we tested the potential non-linearity in the correlation between vitamin D level and the risk of TC through a fixed-effect restricted cubic spline model with four knots at the percentiles of 5, 35, 65, and 95% of the distribution. Then we estimated the linear trend from the correlated ORs and 95% CIs across categories of vitamin D using generalized least squares regression.

Heterogeneity was estimated using *I*^2^ statistics indicating the percentage of heterogeneity that is beyond chance (low heterogeneity: *I*^2^ < 25%; moderate heterogeneity: *I*^2^ = 25–50%; strong heterogeneity: *I*^2^ > 50%). Sensitivity analysis was conducted to evaluated the stability of the results. Finally, we assessed the publication bias through Beggs test and Eggers test.

Results were considered statistically significant when *p*-value was < 0.05, and all statistic analysis were conducted using STATA 14.0 software.

## 3 Results

### 3.1 Study selection, characteristics, and quality assessment

**Figure 1:**
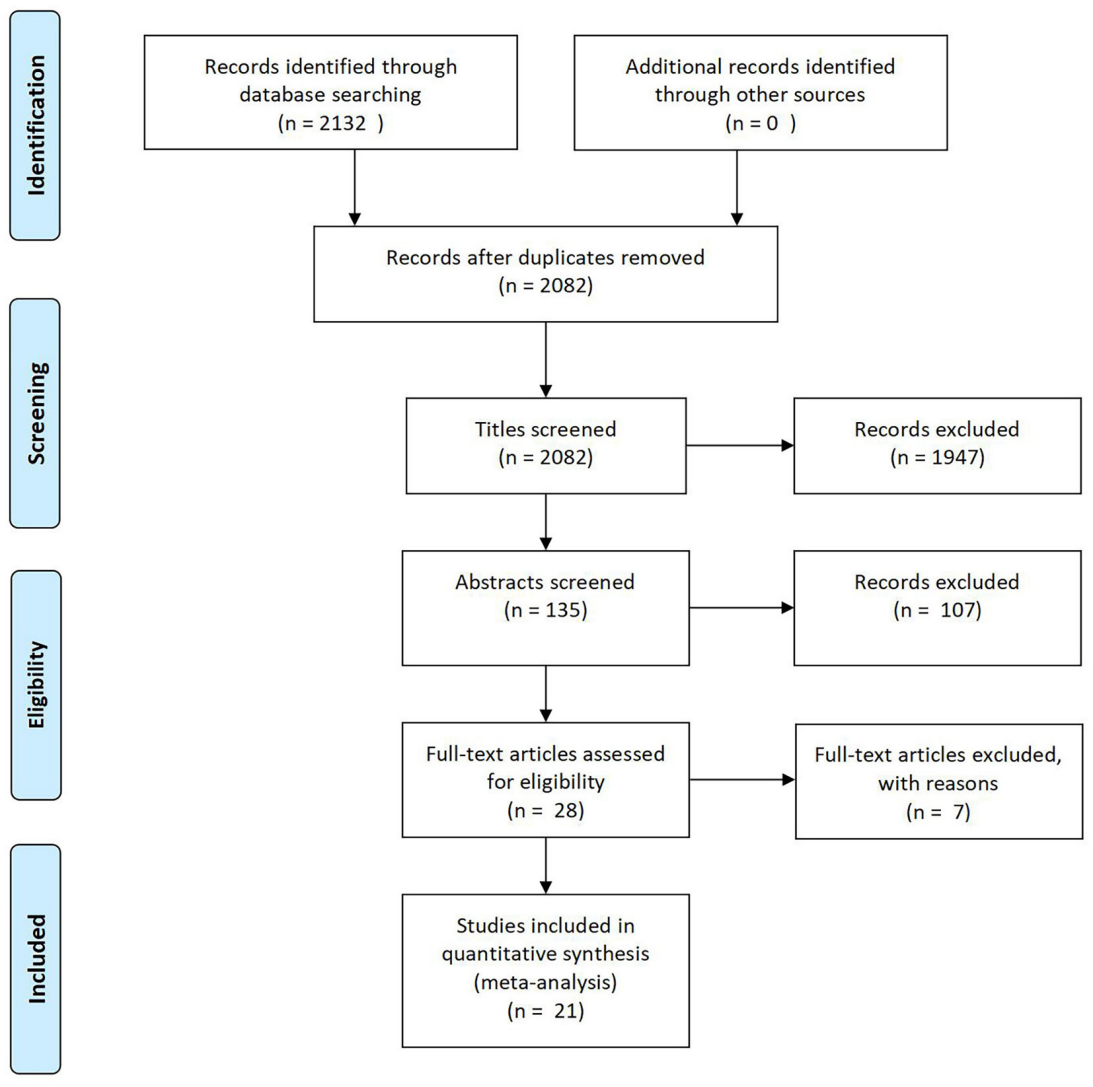
Flow diagram of included studies.

Two thousand one hundred thirty-two articles were yielded through databases searching. After the removal of reduplicates, 2,082 articles remained for the screening of titles. The abstracts of 135 articles were assessed, and finally 28 articles went through full-text screening. Eventually, 21 eligible published articles were included according to the inclusion and exclusion criteria. A flow diagram of study selection is presented in Figure 1.

The included articles were published from 1982 to 2020 and involved 2,434 patients and 7,398 controls, with six of them conducted in China, three from Iran, two from Turkey, and others from Brazil, the United States, Poland, German, Korea, Canada, Romania, Thailand, Japan and Denmark. Sixteen Studies reported the level of vitamin D in cases and controls, 9 reported the OR and 95% CIs, and three reported the dose-response data of vitamin D level and the risk of thyroid cancer. Among all eligible studies, 16 of them were case-control studies, four were cross-sectional studies and one was cohort study. The main characteristics of included studies are shown in Table 2.

**Table 2:** Main characteristics of the included literature.

	**First author**	**Source of control**	**Year**	**Country**	**Study design**	**Type of thyroid cancer**	**Cases**	**controls**	**Methods**
1	Zhang, T	Benign thyroid disease	2018	China	Case-control	Papillary	78	80	ELISA
2	Debora Lucia Seguro Danilovic	Benign thyroid disease	2016	Brazil	Case-control	Papillary and follicular	199	234	CLIA
3	Jacqueline Jonklaas	Benign thyroid disease	2013	United States	Case-control	Papillary and follicular	48	17	LC-MS/MS
4	M.-J. Hu	Health control	2018	China	Case-control	Papillary	138	138	RIA
5	Tomasz Stepien	Health control	2010	Poland	Case-control	Papillary, follocular, anaplastic	50	26	RIA
6	Marissa Penna-Martinez	Health control	2012	German	Case-control	Papillary, follicular	253	302	RIA
7	M RAMEZANI	Health control	2020	Iran	Case-control	Medullary	40	40	ELISA
8	Jie Kuang	Benign thyroid disease	2022	China	Case-control	Papillary	127	128	HPLC
9	Yun Mi Choi	Health control	2015	Korea	Case-control	NA	53	5,133	RIA
10	Qian Song	Health control	2016	China	Case-control	Papillary	62	53	CLIA
11	M.-J. Hu	Health control	2019	China	Case-control	Papillary, follicular, medullary, anaplastic	506	506	RIA
12	S. Yildiz	Benign thyroid disease	2019	Turkey	Case-control	Papillary, medullary, follicular	78	101	CLIA
13	Michael Roskies, BSc	Benign thyroid disease	2012	Canada	Cohort study	NA	42	58	NA
14	Zahra Heidari	Health control	2017	Iran	Case-control	Differentiated	85	85	EIA
15	A. M. Cocolos	Benign thyroid disease	2022	Romania	Cross-sectional	Papillary, follicular	170	200	CLIA
16	Mustafa Sahin	Health control	2013	Turkey	Case-control	Follicular, papillary	344	116	ELISA
17	Zhang, Daqi	Benign thyroid disease	2023	China	Case-control	Papillary	51	49	LS-MS/MS
18	Wiwanitkit, V	Benign thyroid disease	2010	Thailand	Case-control	Papillary, follicular, anaplastic	50	34	EIA
19	Lee, S	Health control	1982	Japan	Cross-sectional	Medullary	8	22	CPBA
20	Emmertsen, K	Health control	1982	Danmark	Cross-sectional	Medullary	12	36	CPBA
21	Emami, Ali	Health control	2017	Iran	Cross-sectional	Medullary	40	40	ELISA

NA, not applicable.

All case-control studies got no < 6 stars and the cohort study got full stars. The results suggested a relatively high quality (Tables 3, 4).

**Table 3:** Quality of included cohort and case-control studies.

**First author**	**Year**	**Study design**	**Selection**	**Comparability**	**Outcome/exposure**	**Total number of stars**
Zhang, T	2018	Case-control	4	0	2	6
Debora Lucia Seguro Danilovic	2016	Case-control	4	2	3	9
Jacqueline Jonklaas	2013	Case-control	2	2	3	7
M.-J. Hu	2018	Case-control	3	2	3	8
Tomasz Stepien	2010	Case-control	3	2	3	8
Marissa Penna-Martinez	2012	Case-control	3	2	3	8
M RAMEZANI	2020	Case-control	3	2	3	8
Jie Kuang	2022	Case-control	3	2	3	8
Yun Mi Choi	2015	Case-control	4	0	3	7
Qian Song	2016	Case-control	3	2	3	8
M.-J. Hu	2019	Case-control	3	2	3	8
S Yildiz	2019	Case-control	3	2	3	8
Michael Roskies, BSc	2012	Cohort study	4	2	3	9
Zahra Heidari	2017	Case-control	3	2	3	8
Mustafa S, ahin	2013	Case-control	3	2	3	8
Zhang, Daqi	2023	Case-control	4	2	2	8
Wiwanitkit, V	2010	Case-control	4	0	2	6

**Table 4:** Quality of included cross-sectional studies.

**First author**	**A.M. Cocolos**	**Lee, S**	**Emmertsen, K**	**Emami, Ali**
Define the source of information	Yes	Yes	No	Yes
List the inclusion and exclusion criteria	Yes	Yes	Yes	Yes
Indicate time period	Yes	Unclear	No	Yes
Indicate whether the subjects were consecutive	Yes	Unclear	Unclear	Unclear
Indicate if evaluators of subjective components were masked	No	No	No	No
Describe any assessment for quality assurance	Yes	Yes	Yes	Yes
Explain any patients exclusions from analysis	Unclear	Unclear	Yes	Yes
Describe how confounding was assessed or controlled	Unclear	No	No	Yes
Explain how missing data were handled	Unclear	Unclear	Unclear	Unclear
Summarize patients response rate	Yes	Unclear	Unclear	Unclear
Clarify what follow-up was expected and percentage of which incomplete data obtained	Unclear	Unclear	Unclear	Unclear

### 3.2 Vitamin D levels

**Figure 2:**
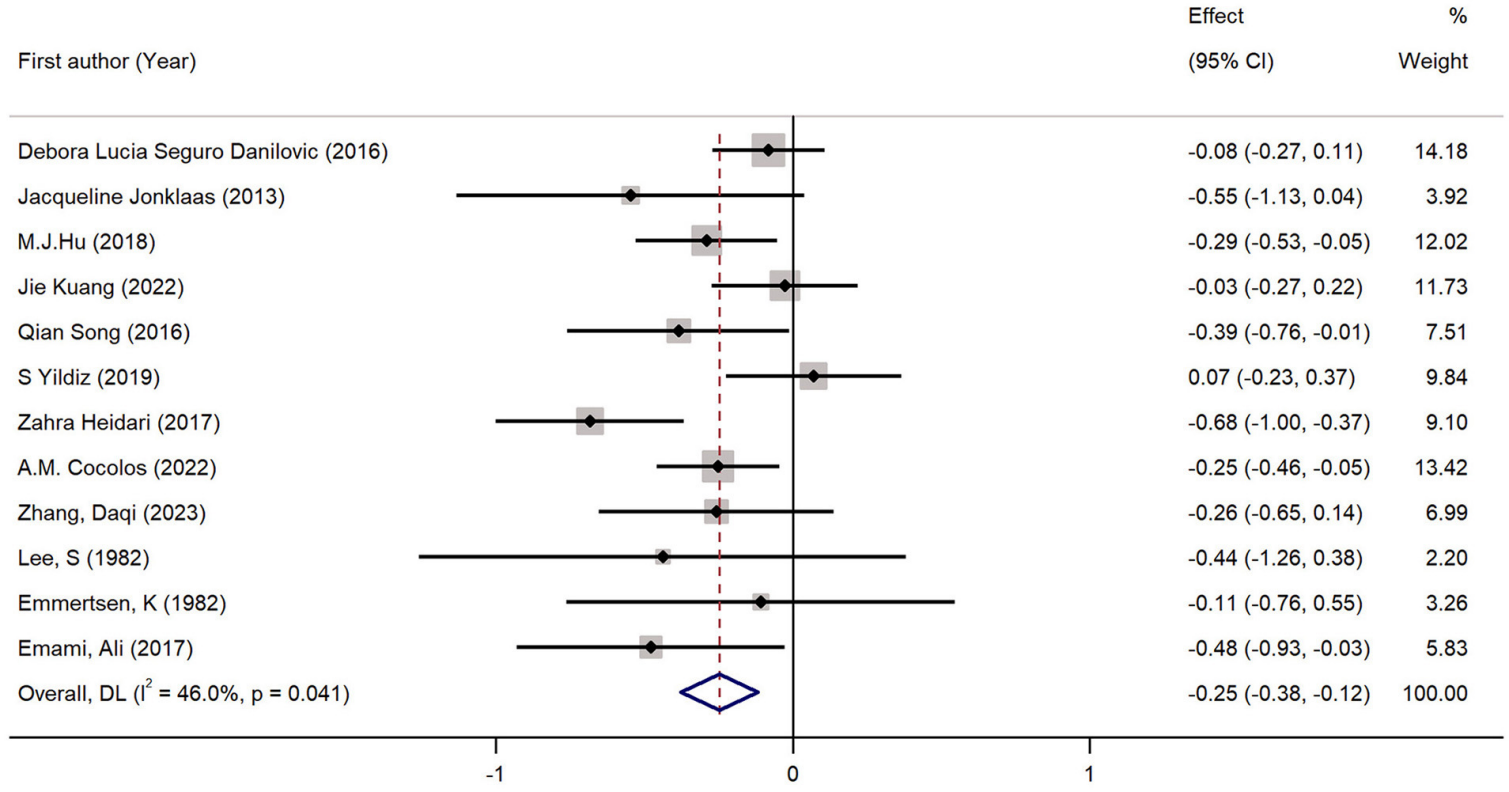
Forest plots and pooled estimates of the effect for the meta-analysis of the standardized mean difference between the 25 (OH)D levels in the patients with thyroid cancer and the controls.

**Figure 3:**
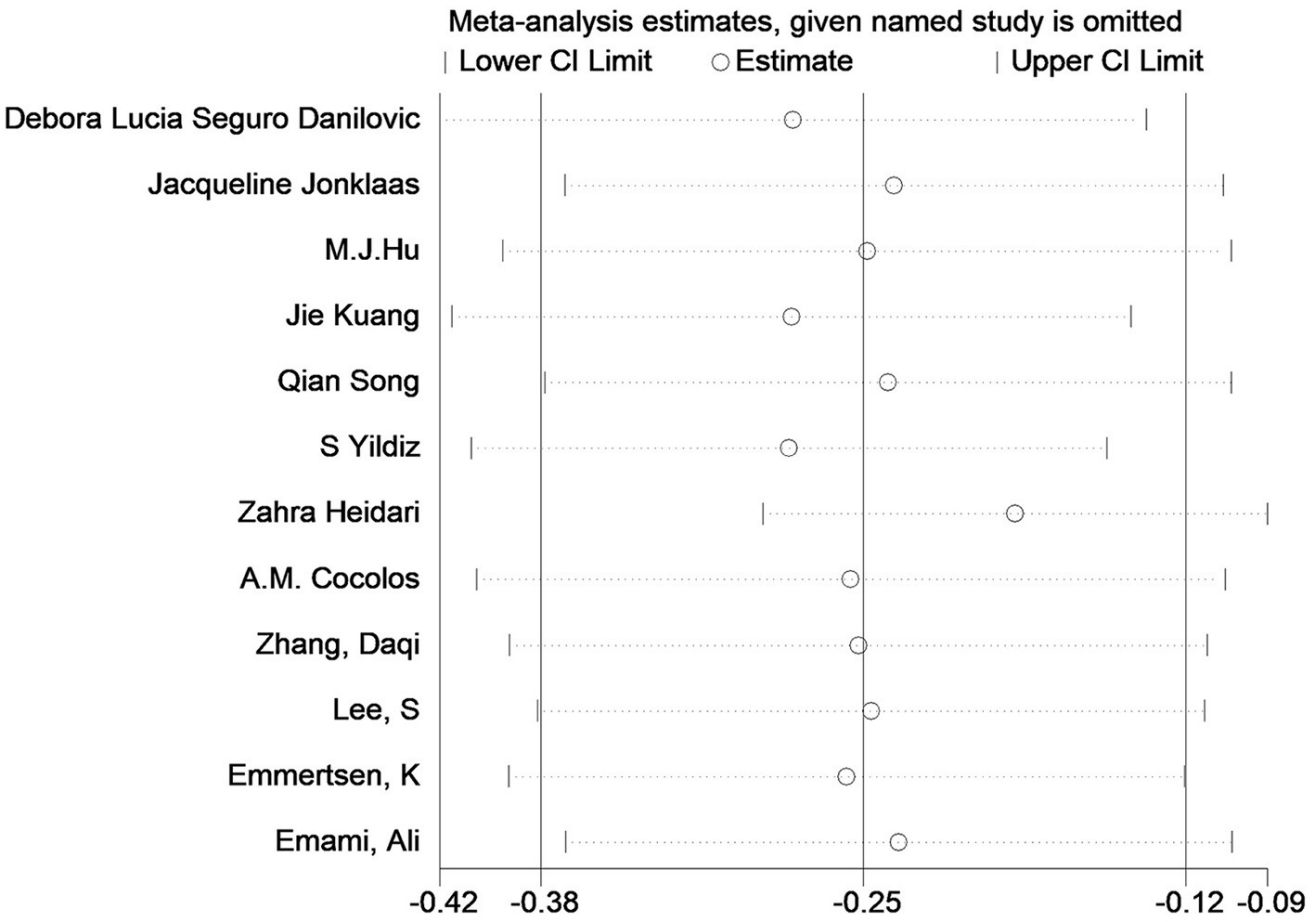
Sensitivity analysis of the standardized mean difference between the 25 (OH)D levels in the patients with thyroid cancer and the controls.

**Figure 4:**
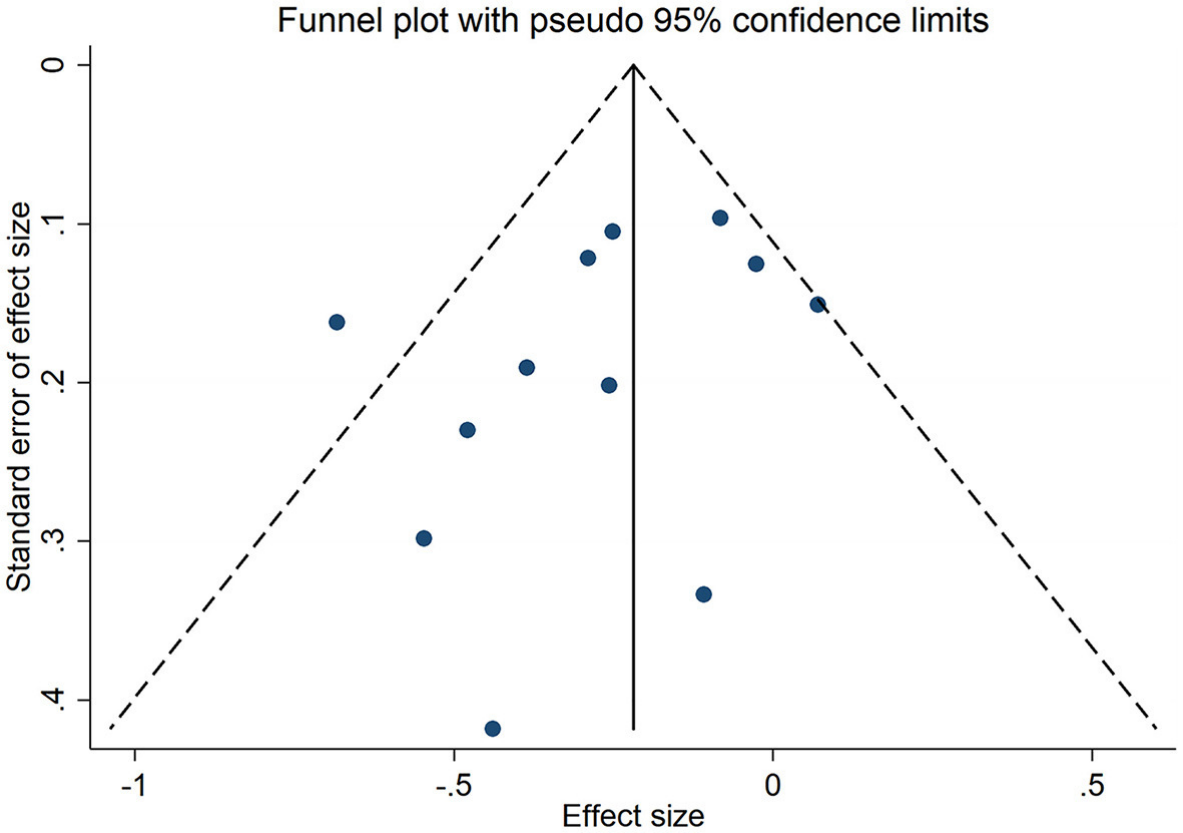
Funnel plot of the studies included in the meta-analysis of the standardized mean difference between the 25 (OH)D levels in the patients with thyroid cancer and the controls.

**Figure 5:**
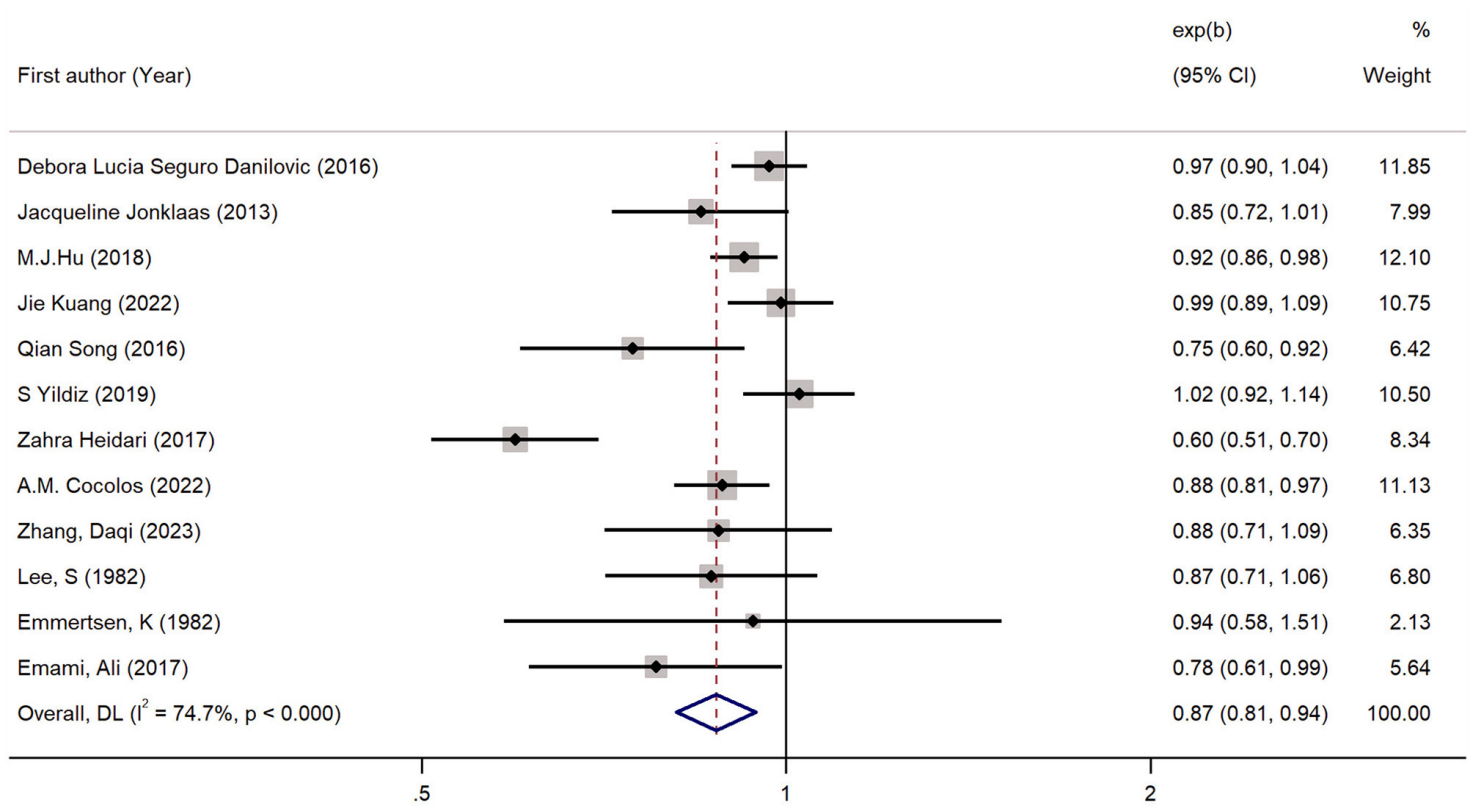
Forest plots and pooled estimates of the effect for the meta-analysis of the ratio of means between the 25 (OH)D levels in the patients with thyroid cancer and the controls.

Twelve studies had evaluated the blood levels of 25 (OH)D in patients with thyroid cancer and controls. The pooled standardized mean difference between the 25 (OH)D levels in TC patients and the controls was −0.25 (95% CI: −0.38, −0.12; *P* < 0.05) with a moderate heterogeneity (*I*^2^ = 46.0%, *P* < 0.05), which indicated that the blood levels of 25 (OH)D in TC patients were significantly lower than those in the control group (Figure 2). To explore the source of heterogeneity, several subgroup analyses were conducted. Table 5 show the results of subgroup analyses based on the testing method of 25 (OH)D, the timing of measurement of 25 (OH)D, the sample source, the countries where the included studies were conducted and the source of controls. The outcomes suggested that the results of this meta-analysis were significant regardless of these factors. Moreover, the heterogeneity of the subgroups using health individuals and individuals with benign thyroid diseases as control groups were 0.0% (*P* = 0.448) and 21.5% (*P* = 0.272), respectively, indicating that the source of controls was a prominent cause of heterogeneity. The results of the meta-regression for each covariate are presented in Table 6, demonstrating that the source of controls (*P* = 0.02) and the age of participants (*P* = 0.009) were the source of the heterogeneity. The results of sensitivity analysis showed that the removal of any single trial did not affect the significance of the outcome (Figure 3). The funnel plot is shown in Figure 4, and the Eggers' and Beggs' tests did not reveal any significant publication bias (*P* = 0.205, *P* = 0.451). And the pooled RoM for level of 25 (OH)D was 0.87 (95% CI: 0.81,0.94; *P* < 0.05; *I*^2^ = 74.7%), which indicated that the 25 (OH)D level in TC cases was 13% lower than that in controls (Figure 5).

**Table 5:** Results of subgroup analysis for the SMD of 25 (OH)D levels.

**Characteristic**	**Subgroup**	**Number of studies**	**SMD (95% CI)**	***I* ^2^**	***P* for heterogeneity**
Overall		12	−0.25 (−0.38,−0.12)	46.0%	0.041
Methods of measurement	CLIA	4	−0.15 (−0.31–0.02)	40.8%	0.167
	LC-MS/MS	2	−0.35 (−0.68,−0.02)	0.0%	0.421
	RIA	1	−0.29 (−0.53,−0.05)	0.0%	< 0.000
	HPLC	1	−0.03 (−0.27,0.22)	0.0%	< 0.000
	EIA	1	−0.68 (−1.00,−0.37)	0.0%	< 0.000
	CPBA	2	−0.24 (−0.75,0.27)	0.0%	0.537
	ELISA	1	−0.48 (−0.93,−0.03)	0.0%	< 0.000
Timing of measurement	Prediagnosis	8	−0.23 (−0.40,−0.06)	61.4%	0.011
	Postdiagnosis	4	−0.32 (−0.51,−0.12)	0.0%	0.797
Sample source	Serum	11	−0.25 (−0.39,−0.10)	49.9%	0.030
	Plasma	1	−0.29 (−0.53,−0.05)	0.0%	< 0.000
Country	Brazil	1	−0.08 (−0.27,0.11)	0.0%	< 0.000
	United States	1	−0.55 (−1.13,0.04)	0.0%	< 0.000
	China	4	−0.21 (−0.37,−0.05)	13.9%	0.323
	Turkey	1	0.07 (−0.23,0.37)	0.0%	< 0.000
	Iran	2	−0.62 (−0.88,−0.36)	0.0%	0.466
	Romania	1	−0.25 (−0.46,−0.05)	0.0%	< 0.000
	Japan	1	−0.44 (−1.26,0.38)	0.0%	< 0.000
	Denmark	1	−0.11 (−0.76,0.55)	0.0%	< 0.000
Source of controls	Benign thyroid disease	6	−0.13 (−0.25,−0.00)	21.5%	0.272
	Health	6	−0.41 (−0.56,−0.26)	0.0%	0.448

**Table 6:** Results of the meta-regression for the level of 25 (OH)D.

**Covariate**	**Coefficient**	**95% confidence interval**	***P*-value**
Methods of measurement	−0.29	(−0.67, 0.09)	0.568
Timing of measurement	0.98	(−0.27, 0.47)	0.568
Country	−0.55	(−1.40, 0.30)	0.149
Sample source	0.04	(−0.47, 0.55)	0.866
Source of controls	0.29	(0.06, 0.52)	0.020
Year	0.00	(−0.15, 0.02)	0.756
Age	0.03	(0.01, 0.04)	0.009
Proportion of women	−0.27	(−1.72, 1.19)	0.691

**Figure 6:**
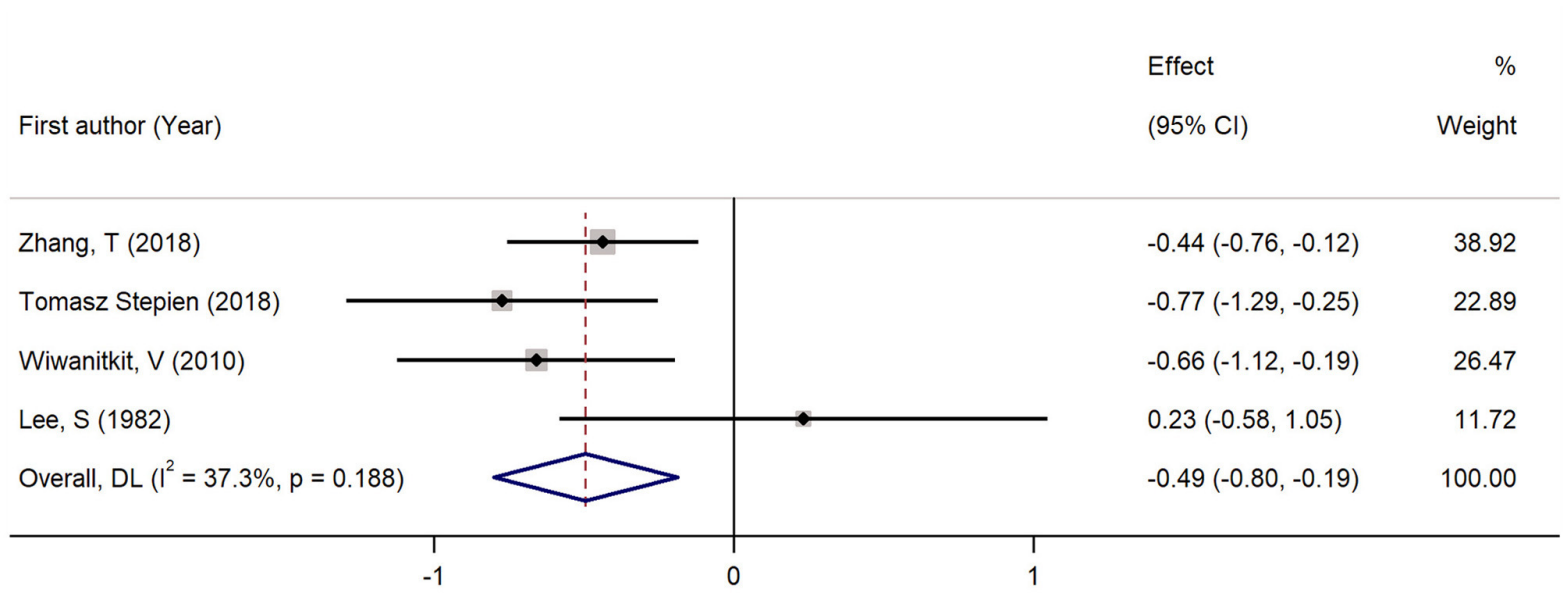
Forest plots and pooled estimates of the effect for the meta-analysis of the standardized mean difference between the 1, 25 (OH)D levels in the patients with thyroid cancer and the controls.

**Figure 7:**
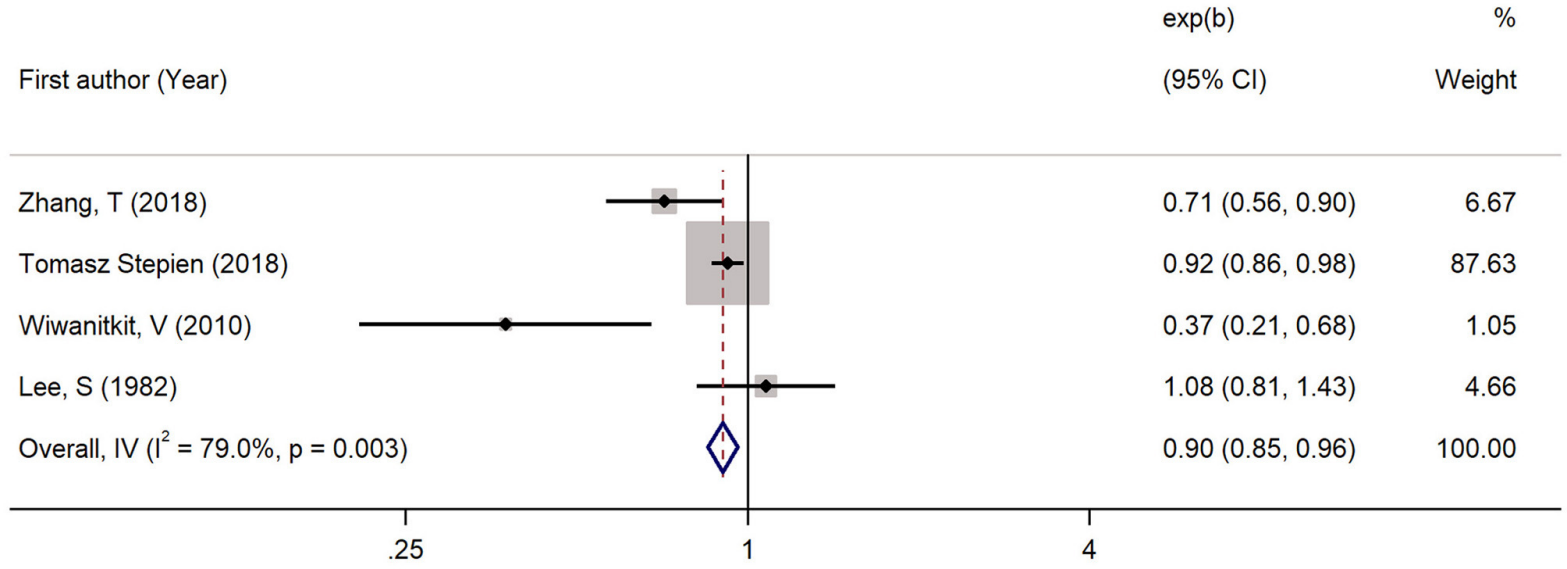
Forest plots and pooled estimates of the effect for the meta-analysis of the ratio of means between the 1, 25 (OH)D levels in the patients with thyroid cancer and the controls.

Four studies had reported the blood levels of 1,25 (OH)D in cases with TC and controls. As shown in Figure 6, the pooled standardized mean difference was −0.49 (95% CI: −0.80, −0.19; *P* < 0.05) with a heterogeneity of 37.3% (*P* = 0.188). The pooled RoM was 0.90 (95% CI: 0.85, 0.96; *P* < 0.05; *I*^2^ = 79.0%), demonstrating that the 1,25 (OH)D level in TC cases were 10% lower than in controls (Figure 7).

### 3.3 The association between vitamin D deficiency and the risk of TC

**Figure 8:**
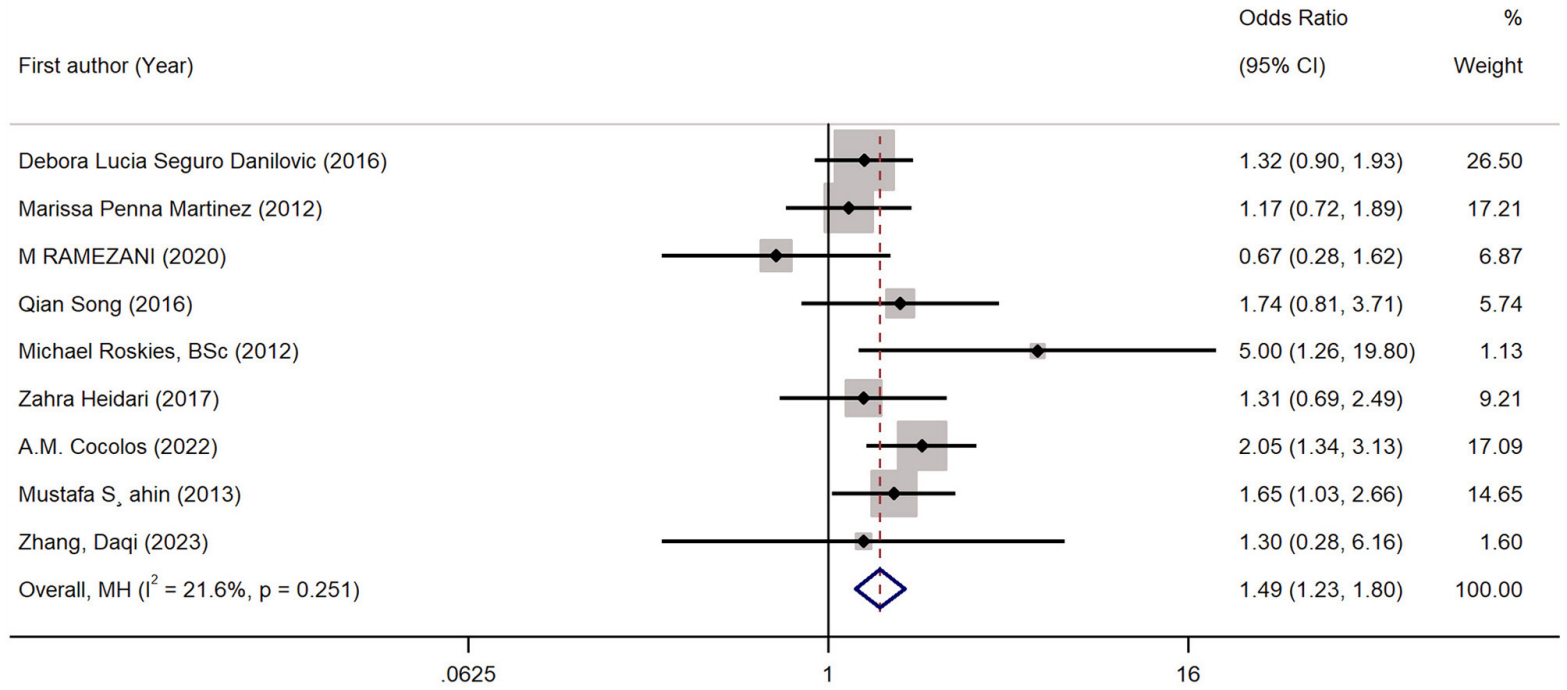
Forest plots and pooled estimates of the effect for the meta-analysis of the association between vitamin D deficiency and the risk of thyroid cancer.

**Figure 9:**
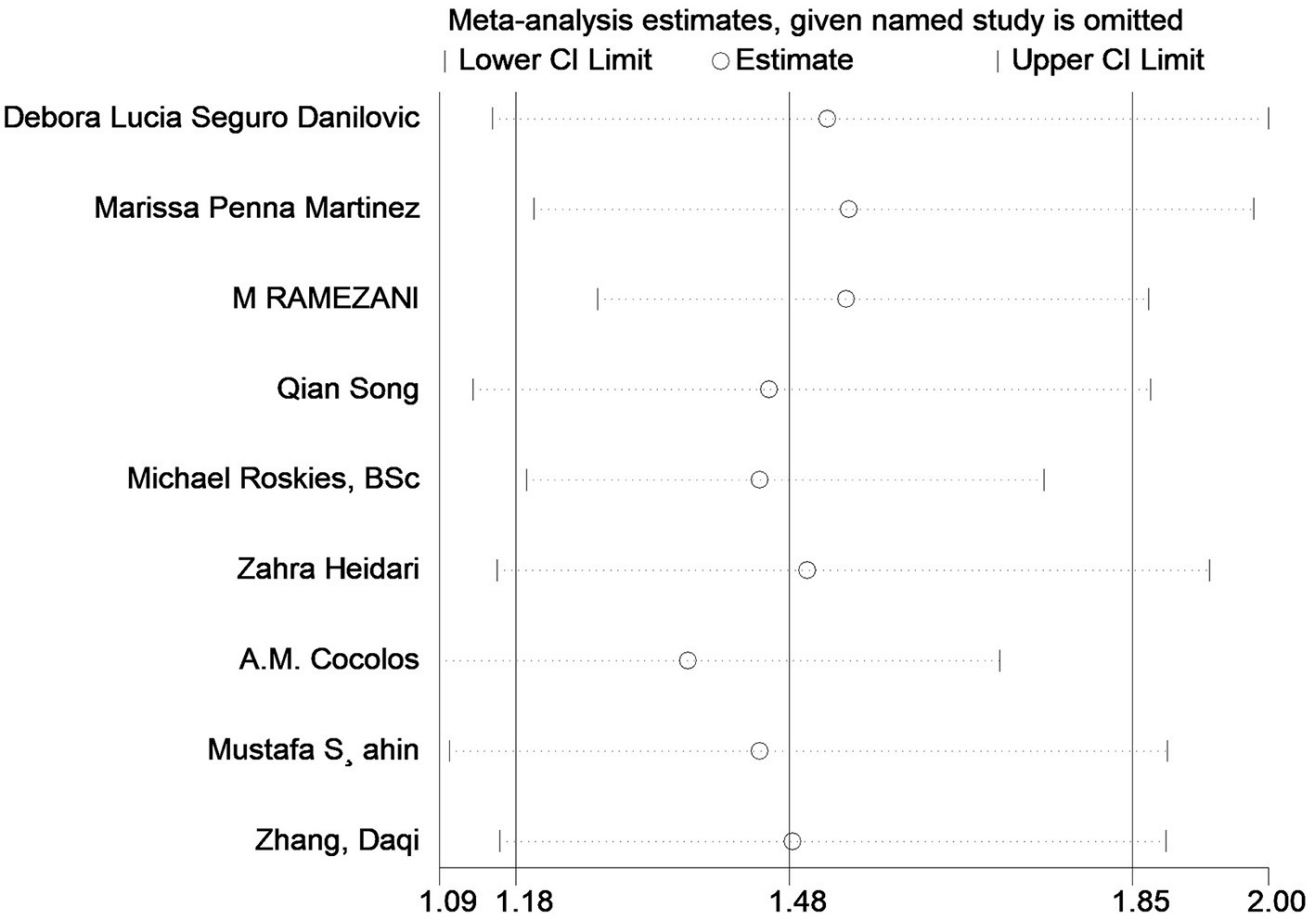
Sensitivity analysis of the association between vitamin D deficiency and the risk of thyroid cancer.

This meta-analysis included nine studies, demonstrating that vitamin D deficiency was associated with a higher risk of TC (pooled OR: 1.49, 95% CI: 1.23, 1.80; *P* < 0.05) (Figure 8). And the heterogeneity was not significant (*I*^2^ = 21.6%, *P* = 0.251). Subgroup analyses demonstrated that the result of the meta-analysis was significant regardless of whether the source of controls were healthy individuals or patients with benign thyroid disease (Table 7). However, the subgroup whose vitamin D levels were measured before the diagnosis of TC showed an insignificant results, which might be due to the limited number of trials included in the subgroup (Table 7). The results of meta regression are shown in Table 8, and the sensitivity analysis confirmed the stability of this meta-analysis (Figure 9). The publication bias was not significant according to the results of Eggers and Beggs tests (*P* = 0.394, *P* = 0.917).

**Table 7:** Results of subgroup analysis for the association between vitamin D deficiency and the risk of thyroid cancer.

**Characteristic**	**Subgroup**	**Number of studies**	**OR (95% CI)**	***I* ^2^**	***P* for heterogeneity**
Overall		9	1.49 (1.23, 1.80)	21.6%	0.251
Source of controls	Benign thyroid disease	4	1.76 (1.15, 2.68)	39.1%	0.177
	Health controls	5	1.33 (1.02, 1.73)	0.0%	0.420
Timing of measurement	Prediagnosis	7	1.63 (1.31, 2.01)	0.0%	0.490
	Postdiagnosis	2	1.00 (0.62, 1.63)	15.6%	0.276

**Table 8:** Results of the meta-regression for the association between vitamin D deficiency and the risk of thyroid cancer.

**Covariate**	**Coefficient**	**95% confidence interval**	***P*-value**
Timing of measurement	0.14	(−0.45, 0.73)	0.587
Source of controls	0.26	(−0.29, 0.81)	0.302

### 3.4 Dose-response meta-analysis

**Figure 10:**
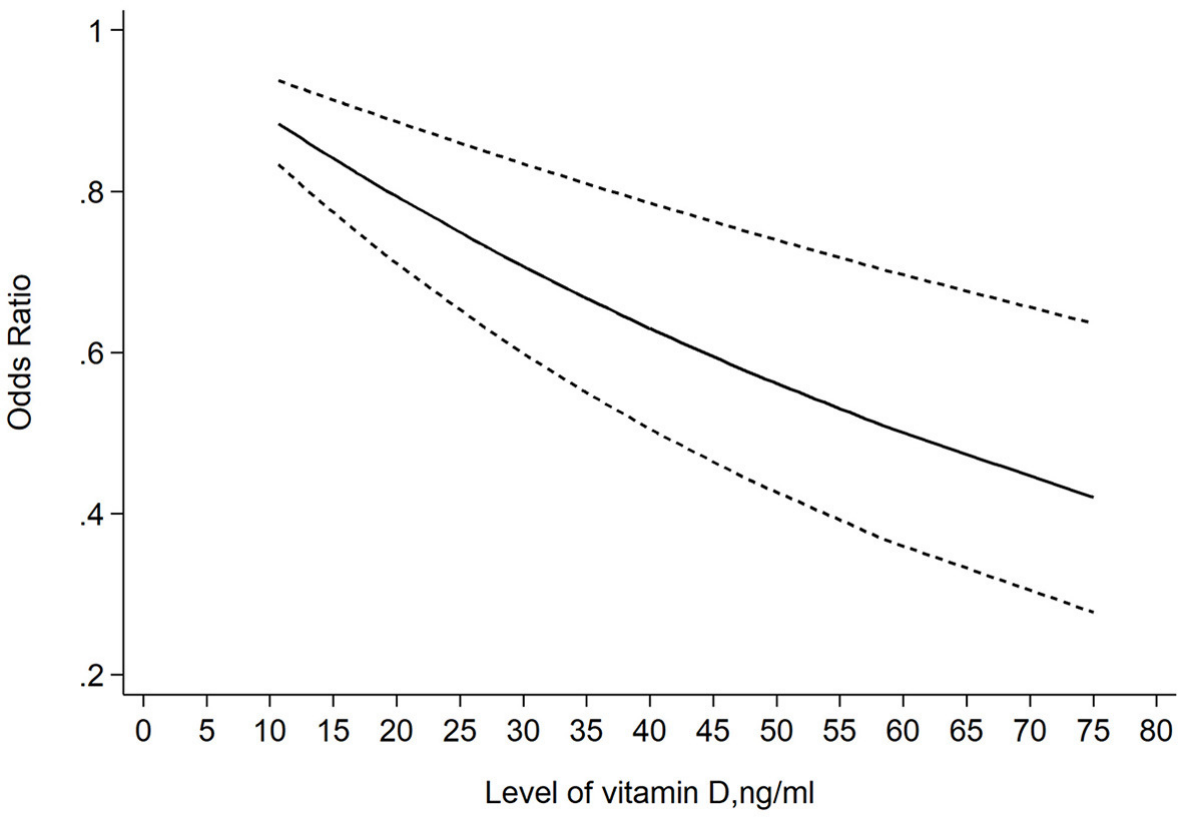
Dose-response relationship between 25 (OH)D levels and the odds ratio of thyroid cancer.

Finally, we evaluated the dose-response relationship between the level of blood 25 (OH)D and the risk of TC. As shown in Figure 10, a significant linear correlation between blood 25 (OH)D level and the risk of TC. For each 10 ng/ml decrease in 25 (OH)D levels, there is a 6% increase in the risk of TC (OR: 0.94; 95% CI: 0.89, 0.99).

## 4 Discussion

In the past few years, vitamin D has attracted clinical interest for its association with cancers. Several observational studies have demonstrated a correlation between vitamin D deficiency and the occurrence and fatality of various types of cancer (28–35). Additionally, laboratory investigations have provided substantial evidence supporting the anticancer properties of vitamin D through vitamin D receptor binding or indirect interaction with transcriptional regulators and cell signally systems (36–38). Specifically, vitamin D has demonstrated the ability to impede the expression of proto-oncogene c-MYC, while concurrently promoting the accumulation of p27. Consequently, this regulation of the cell cycle ultimately leads to the inhibition of cellular proliferation (39–41). Furthermore, vitamin D has been observed to enhance the expression of fibronectin via the PTEN/PI3 kinase pathway, as well as reverse the cadherin switch. As a result, this leads to a reduction in the invasion and metastasis of cancer cells (42, 43). In addition, the potential of vitamin D to improve the inflammatory micro-environment in TC by inhibiting the proliferation of Th1 and Th17 as well as the expression of cytokines such as IL-1, IL-17 and IL-21 has been reported (44, 45). Although preclinical trials have revealed the potential anticancer effect of vitamin D and that vitamin D receptor polymorphisms are likely to be closely correlated with the risk of TC, data from human remain to be inconsistent (6). A number of studies have suggested that vitamin D level is unreliable as a risk and prognostic factor for TC, while others have linked vitamin D status to the risk of TC (46–49). Thus, a meta-analysis is necessary to comprehensively analyze existing clinical evidence and draw a conclusion. Despite 2 relative meta-analyses published, new clinical evidence has been published in the last few years (24, 25). Thus, we updated the included literature and performed a completely new meta-analysis. Our analysis reported the difference between the levels of 1,25 (OH)D in TC cases and controls for the first time. Additionally, we introduced RoM, a new effect size, to determine the difference between vitamin levels in TC cases and the controls, so that the disparity in vitamin D levels between groups can be easily demonstrated in percentage terms, which was easier to clinically interpret compared to SMD, a classical effect size of meta-analysis for continuous data. (50). As far as we know, our study was the first to conduct a dose-response meta-analysis to reveal the relationship between the status of vitamin D and the risk of TC.

Based on 21 trials involving 2,434 patients with TC and 7,398 controls, our meta-analysis indicated that the blood level of 25 (OH)D in TC cases was significantly lower than that in healthy individuals or those with benign thyroid diseases. This outcome did not differ by the testing methods of 25 (OH)D, countries where the studies conducted, the timing of 25 (OH)D measurement, the source of samples, and the source of the controls, which was consistent with the results of the previous meta-analysis. Subgroup analysis and meta regression indicated that the source of controls were the major source of the heterogeneity, which was noteworthy. Actually, we found a few articles regarding relatively low vitamin D status in patients with benign thyroid disease such as thyroid nodules (51, 52). Regrettably, studies on the mechanism linking vitamin D and thyroid disease are still primarily focused on autoimmune thyroiditis and thyroid cancer, with other benign thyroid diseases receiving inadequate attention. Thus, more experimental evidence is urgently needed to clarify the mechanism lying behind the results of our subgroup analysis. Nevertheless, we still found the level of 25 (OH)D in TC cases significant lower than cases with benign thyroid disease, which make the level of 25 (OH)D still a potential and valuable risk factor for TC. Moreover, according to the results of meta-regression, the age of participants was another source of the heterogeneity, which was not surprising since cumulative studies had reported the effect of age on serum vitamin D level and on the capacity of human skin to produce vitamin D (53, 54).

We first reported that the level of 25 (OH)D in cases with TC was likely to be 13% lower than in controls. And our study was the first meta-analysis reporting the level of 1, 25 (OH)D, the hormonally active form of vitamin D, in TC cases. As shown in our article, the blood level of 1,25 (OH)D was relatively low and might be 10% lower than that in controls, which was not mentioned in any previous meta-analysis. The outcome of our meta-analysis had also demonstrated that vitamin D deficiency might potentially increase the risk of TC by 49%. This association was significant in subgroups of both healthy controls and benign thyroid disease. Although it was not significant in the subgroup whose vitamin D levels were measured before the diagnosis of TC, we considered the reliability of this result to be limited. Because only two trials were included in this subgroup which could lead to a false negative outcome from a statistic standpoint. Finally, our study firstly conducted a dose-response meta-analysis between the level of vitamin D and the risk of TC. Our results showed an inverse linear association between 25 (OH)D level and the TC risk and 10 ng/ml of increase in 25 (OH)D level was correlated with 6% lower risk of TC.

Several limitations of this meta-analysis warrant attention. First, the majority of the included studies were case-control studies, thereby posing challenges in establishing a definitive causal relationship between vitamin D levels and the risk of TC. Therefore, more prospective cohort studies on a larger scale were expected to improve the reliability of our analysis. Secondly, the standards of vitamin D deficiency were inconsistent, which was likely to influence the results of meta-analysis adopting OR as the effect size. Third, several studies included cases with multiple pathological TC types and lacked the necessary data of cases with each kind of TC, which created a barrier to performing a subgroup analysis based on types of TC. Forth, we failed to explore the impact of multiple factors including body-mass index, dietary habits and smoking on the results of the meta-analyses due to the absence of data. Thus, more articles published with complete data would be needed in the future to address these issues.

## 5 Conclusion

The findings of our study indicate that individuals with TC have lower blood levels of 25 (OH)D and 1,25 (OH)D compared to controls. Additionally, an inverse linear correlation between 25 (OH)D level and the risk of TC is existed. Thus, although numerous negative results have been published, we still propose vitamin D deficiency as a potential risk factor for TC. Furthermore, robust clinical and preclinical evidence is needed for a large, well-executed and more inclusive systematic review with meta-analysis on this topic.

## Data availability statement

The original contributions presented in the study are included in the article/supplementary material, further inquiries can be directed to the corresponding author.

## Author contributions

YH: Data curation, Formal analysis, Investigation, Methodology, Software, Writing – original draft, Writing – review & editing. CX: Conceptualization, Supervision, Validation, Writing – original draft, Writing – review & editing. SR: Data curation, Methodology, Writing – review & editing. LD: Data curation, Methodology, Writing – review & editing. JG: Data curation, Software, Writing – review & editing. XL: Data curation, Investigation, Software, Supervision, Writing – original draft, Writing – review & editing.

## References

[1] Filetti S Durante C Hartl D Leboulleux S Locati LD Newbold K . Thyroid cancer: ESMO clinical practice guidelines for diagnosis, treatment and follow-up†. Ann Oncol. (2019) 30:1856–83. 10.1093/annonc/mdz400 31549998

[2] Raue F Frank-Raue K. Thyroid cancer: risk-stratified management and individualized therapy. Clin Cancer Res. (2016) 22:5012–21. 10.1158/1078-0432.CCR-16-0484 27742787

[3] Sung H Ferlay J Siegel RL Laversanne M Soerjomataram I Jemal A . Global cancer statistics 2020: GLOBOCAN estimates of incidence and mortality worldwide for 36 cancers in 185 countries. CA Cancer J Clin. (2021) 71:209–49. 10.3322/caac.21660 33538338

[4] Lim H Devesa SS Sosa JA Check D Kitahara CM. Trends in thyroid cancer incidence and mortality in the United States, 1974-2013. JAMA. (2017) 317:1338–48. 10.1001/jama.2017.2719 28362912 PMC8216772

[5] Kitahara CM Sosa JA. The changing incidence of thyroid cancer. Nat Rev Endocrinol. (2016) 12:646–53. 10.1038/nrendo.2016.110 27418023 PMC10311569

[6] Penna-Martinez M Ramos-Lopez E Stern J Hinsch N Hansmann ML Selkinski I . Vitamin d receptor polymorphisms in differentiated thyroid carcinoma. Thyroid. (2009) 19:623–28. 10.1089/thy.2008.0388 19499989

[7] Pizzato M Li M Vignat J Laversanne M Singh D La Vecchia C . The epidemiological landscape of thyroid cancer worldwide: GLOBOCAN estimates for incidence and mortality rates in 2020. Lancet Diabetes Endocrinol. (2022) 10:264–72. 10.1016/S2213-8587(22)00035-3 35271818

[8] Li M Dal Maso L Vaccarella S. Global trends in thyroid cancer incidence and the impact of overdiagnosis. Lancet Diabetes Endocrinol. (2020) 8:468–70. 10.1016/S2213-8587(20)30115-7 32445733

[9] Miranda-Filho A Lortet-Tieulent J Bray F Cao B Franceschi S Vaccarella S . Thyroid cancer incidence trends by histology in 25 countries: a population-based study. Lancet Diabetes Endocrinol. (2021) 9:225–34. 10.1016/S2213-8587(21)00027-9 33662333

[10] Chen DW Lang B McLeod D Newbold K Haymart MR. Thyroid cancer. Lancet. (2023) 401:1531–44. 10.1016/S0140-6736(23)00020-X 37023783

[11] Chen J Wang C Shao B. Global, regional, and national thyroid cancer age-period-cohort modeling and BAYESIAN predictive modeling studies: a systematic analysis of the Global Burden of Disease Study 2019. Heliyon. (2023) 9:e22490. 10.1016/j.heliyon.2023.e22490 38045179 PMC10689957

[12] Laha D Nilubol N Boufraqech M. New therapies for advanced thyroid cancer. Front Endocrinol. (2020) 11:82. 10.3389/fendo.2020.00082 32528402 PMC7257776

[13] Alexander EK Cibas ES. Diagnosis of thyroid nodules. Lancet Diabetes Endocrinol. (2022) 10:533–39. 10.1016/S2213-8587(22)00101-2 35752200

[14] Xing M. Molecular pathogenesis and mechanisms of thyroid cancer. Nat Rev Cancer. (2013) 13:184–99. 10.1038/nrc3431 23429735 PMC3791171

[15] Nikiforov YE Nikiforova MN. Molecular genetics and diagnosis of thyroid cancer. Nat Rev Endocrinol. (2011) 7:569–80. 10.1038/nrendo.2011.142 21878896

[16] Schlumberger M Leboulleux S. Current practice in patients with differentiated thyroid cancer. Nat Rev Endocrinol. (2021) 17:176–88. 10.1038/s41574-020-00448-z 33339988

[17] Gallagher JC Rosen CJ. Vitamin D: 100 years of discoveries, yet controversy continues. Lancet Diabetes Endocrinol. (2023) 11:362–74. 10.1016/S2213-8587(23)00060-8 37004709

[18] Christakos S Dhawan P Verstuyf A Verlinden L Carmeliet G. Vitamin D: metabolism, molecular mechanism of action, and pleiotropic effects. Physiol Rev. (2016) 96:365–408. 10.1152/physrev.00014.2015 26681795 PMC4839493

[19] Palanca A Ampudia-Blasco FJ Real JT. The controversial role of vitamin D in thyroid cancer prevention. Nutrients. (2022) 14:2593. 10.3390/nu14132593 35807774 PMC9268358

[20] Bains A Mur T Wallace N Noordzij JP. The role of vitamin D as a prognostic marker in papillary thyroid cancer. Cancers. (2021) 13:143516. 10.3390/cancers13143516 34298730 PMC8304998

[21] Hu MJ Niu QS Wu HB Lu XL Wang L Tong XR . Association of thyroid cancer risk with plasma 25-hydroxyvitamin D and vitamin D binding protein: a case-control study in china. J Endocrinol Invest. (2020) 43:799–808. 10.1007/s40618-019-01167-7 31863361

[22] Jonklaas J Danielsen M Wang H. A pilot study of serum selenium, vitamin D, and thyrotropin concentrations in patients with thyroid cancer. Thyroid. (2013) 23:1079–86. 10.1089/thy.2012.0548 23350941 PMC3770246

[23] Barrea L Gallo M Ruggeri RM Giacinto PD Sesti F Prinzi N . Nutritional status and follicular-derived thyroid cancer: an update. Crit Rev Food Sci Nutr. (2021) 61:25–59. 10.1080/10408398.2020.1714542 31997660

[24] Hu MJ Zhang Q Liang L Wang SY Zheng XC Zhou MM . Association between vitamin d deficiency and risk of thyroid cancer: a case-control study and a meta-analysis. J Endocrinol Invest. (2018) 41:1199–210. 10.1007/s40618-018-0853-9 29464660

[25] Zhao J Wang H Zhang Z Zhou X Yao J Zhang R . Vitamin D deficiency as a risk factor for thyroid cancer: a meta-analysis of case-control studies. Nutrition. (2019) 57:5–11. 10.1016/j.nut.2018.04.015 30086436

[26] Wan X Wang W Liu J Tong T. Estimating the sample mean and standard deviation from the sample size, median, range and/or interquartile range. Bmc Med Res Methodol. (2014) 14:135. 10.1186/1471-2288-14-135 25524443 PMC4383202

[27] Luo D Wan X Liu J Tong T. Optimally estimating the sample mean from the sample size, median, mid-range, and/or mid-quartile range. Stat Methods Med Res. (2018) 27:1785–805. 10.1177/0962280216669183 27683581

[28] Kim Y Chang Y Cho Y Chang J Kim K Park DI . Serum 25-hydroxyvitamin d levels and risk of colorectal cancer: an age-stratified analysis. Gastroenterology. (2023) 165:920–31. 10.1053/j.gastro.2023.06.029 37429364

[29] Lawler T Warren AS. Serum 25-hydroxyvitamin D and cancer risk: a systematic review of Mendelian randomization studies. Nutrients. (2023) 15:422. 10.3390/nu15020422 36678292 PMC9865859

[30] Wang QL Ma C Yuan C Shi Q Wolpin BM Zhang Y . Plasma 25-hydroxyvitamin d levels and survival in stage III colon cancer: findings from CALGB/SWOG 80702 (alliance). Clin Cancer Res. (2023) 29:2621–30. 10.1158/1078-0432.CCR-23-0447 37289007 PMC10524689

[31] Virtanen JK Nurmi T Aro A Bertone-Johnson ER Hyppönen E Kröger H . Vitamin D supplementation and prevention of cardiovascular disease and cancer in the finnish vitamin D trial: a randomized controlled trial. Am J Clin Nutr. (2022) 115:1300–10. 10.1093/ajcn/nqab419 34982819 PMC9071497

[32] Trummer O Langsenlehner U Krenn-Pilko S Pieber TR Obermayer-Pietsch B Gerger A . Vitamin D and prostate cancer prognosis: a Mendelian Randomization Study. World J Urol. (2016) 34:607–11. 10.1007/s00345-015-1646-9 26209090

[33] Limonte CP Zelnick LR Hoofnagle AN Thadhani R Melamed ML Mora S . Effects of vitamin D(3) supplementation on cardiovascular and cancer outcomes by EGFR in vital. Kidney360. (2022) 3:2095–105. 10.34067/KID.0006472022 36591342 PMC9802543

[34] Chandler PD Buring JE Manson JE Giovannucci EL Moorthy MV Zhang S . Circulating vitamin D levels and risk of colorectal cancer in women. Cancer Prev Res. (2015) 8:675–82. 10.1158/1940-6207.CAPR-14-0470 25813525 PMC4526335

[35] Feldman D Krishnan AV Swami S Giovannucci E Feldman BJ. The role of vitamin D in reducing cancer risk and progression. Nat Rev Cancer. (2014) 14:342–57. 10.1038/nrc3691 24705652

[36] Manson JE Bassuk SS Buring JE. Vitamin D, calcium, and cancer: approaching daylight? JAMA. (2017) 317:1217–18. 10.1001/jama.2017.2155 28350909

[37] Rozmus D Ciesielska A Płomiński J Grzybowski R Fiedorowicz E Kordulewska N . Vitamin D binding protein (VDBP) and its gene polymorphisms-the risk of malignant tumors and other diseases. Int J Mol Sci. (2020) 21:217822. 10.3390/ijms21217822 33105665 PMC7659952

[38] Roehlen N Doering C Hansmann ML Gruenwald F Vorlaender C Bechstein WO . Vitamin D, FOXO3A, and Sirtuin1 in Hashimoto's thyroiditis and differentiated thyroid cancer. Front Endocrinol. (2018) 9:527. 10.3389/fendo.2018.00527 30271381 PMC6142903

[39] Kim D. The role of vitamin D in thyroid diseases. Int J Mol Sci. (2017) 18:91949. 10.3390/ijms18091949 28895880 PMC5618598

[40] Salehi-Tabar R Nguyen-Yamamoto L Tavera-Mendoza LE Quail T Dimitrov V An BS . Vitamin D receptor as a master regulator of the c-MYC/MXD1 network. Proc Natl Acad Sci USA. (2012) 109:18827–32. 10.1073/pnas.1210037109 23112173 PMC3503153

[41] Chiang KC Kuo SF Chen CH Ng S Lin SF Yeh CN . MART-10, the vitamin d analog, is a potent drug to inhibit anaplastic thyroid cancer cell metastatic potential. Cancer Lett. (2015) 369:76–85. 10.1016/j.canlet.2015.07.024 26282787

[42] Liu W Asa SL Fantus IG Walfish PG Ezzat S. Vitamin d arrests thyroid carcinoma cell growth and induces p27 dephosphorylation and accumulation through PTEN/Akt-dependent and -independent pathways. Am J Pathol. (2002) 160:511–19. 10.1016/S0002-9440(10)64870-5 11839571 PMC1850654

[43] Liu W Asa SL Ezzat S. 1alpha,25-Dihydroxyvitamin D3 targets pten-dependent fibronectin expression to restore thyroid cancer cell adhesiveness. Mol Endocrinol. (2005) 19:2349–57. 10.1210/me.2005-0117 15890670

[44] Mele C Caputo M Bisceglia A Samà MT Zavattaro M Aimaretti G . Immunomodulatory effects of vitamin D in thyroid diseases. Nutrients. (2020) 12:1444. 10.3390/nu12051444 32429416 PMC7284826

[45] Xi C Zhang GQ Sun ZK Song HJ Shen CT Chen XY . Interleukins in thyroid cancer: from basic researches to applications in clinical practice. Front Immunol. (2020) 11:1124. 10.3389/fimmu.2020.01124 32655554 PMC7325887

[46] Kuang J Jin Z Chen L Zhao Q Huang H Liu Z . Serum 25-hydroxyvitamin D level is unreliable as a risk factor and prognostic marker in papillary thyroid cancer. Ann Transl Med. (2022) 10:193. 10.21037/atm-22-10 35280388 PMC8908184

[47] Demircioglu ZG Aygun N Demircioglu MK Ozguven BY Uludag M. Low vitamin D status is not associated with the aggressive pathological features of papillary thyroid cancer. Sisli Etfal Hastan Tip Bul. (2022) 56:132–36. 10.14744/SEMB.2022.36048 35515959 PMC9040301

[48] Choi YM Kim WG Kim TY Bae SJ Kim HK Jang EK . Serum vitamin D3 levels are not associated with thyroid cancer prevalence in euthyroid subjects without autoimmune thyroid disease. Korean J Intern Med. (2017) 32:102–08. 10.3904/kjim.2015.090 27581957 PMC5214716

[49] Ahn HY Chung YJ Park KY Cho BY. Serum 25-hydroxyvitamin D level does not affect the aggressiveness and prognosis of papillary thyroid cancer. Thyroid. (2016) 26:429–33. 10.1089/thy.2015.0516 26739552

[50] Friedrich JO Adhikari NK Beyene J. Ratio of means for analyzing continuous outcomes in meta-analysis performed as well as mean difference methods. J Clin Epidemiol. (2011) 64:556–64. 10.1016/j.jclinepi.2010.09.016 21447428

[51] Bolat H Erdogan A. Benign nodules of the thyroid gland and 25-hydroxy-vitamin D levels in euthyroid patients. Ann Saudi Med. (2022) 42:83–8. 10.5144/0256-4947.2022.83 35380060 PMC8982002

[52] Laney N Meza J Lyden E Erickson J Treude K Goldner W. The prevalence of vitamin d deficiency is similar between thyroid nodule and thyroid cancer patients. Int J Endocrinol. (2010) 2010:805716. 10.1155/2010/805716 20016683 PMC2779458

[53] van der Wielen RP Löwik MR van den Berg H de Groot LC Haller J Moreiras O . Serum vitamin D concentrations among elderly people in Europe. Lancet. (1995) 346:207–10. 10.1016/S0140-6736(95)91266-5 7616799

[54] MacLaughlin J Holick MF. Aging decreases the capacity of human skin to produce vitamin D3. J Clin Invest. (1985) 76:1536–38. 10.1172/JCI112134 2997282 PMC424123

